# Co-utilization of glycerol and lignocellulosic hydrolysates enhances anaerobic 1,3-propanediol production by *Clostridium diolis*

**DOI:** 10.1038/srep19044

**Published:** 2016-01-11

**Authors:** Bo Xin, Yu Wang, Fei Tao, Lixiang Li, Cuiqing Ma, Ping Xu

**Affiliations:** 1 State Key Laboratory of Microbial Technology, Shandong University, Jinan 250100, PR China; 2 State Key Laboratory of Microbial Metabolism, and School of Life Sciences & Biotechnology, Shanghai Jiao Tong University, Shanghai 200240, PR China; 3 Joint International Research Laboratory of Metabolic & Developmental Sciences, Shanghai Jiao Tong University, Shanghai 200240, PR China; 4 Shanghai Collaborative Innovation Center for Biomanufacturing, East China University of Science and Technology, Shanghai 200237, PR China

## Abstract

Anaerobic fermentation using lignocellulosic hydrolysates as co-substrates is an economically attractive method to enhance 1,3-propanediol (1,3-PD) production by increasing the conversion yield from glycerol. Lignocellulosic hydrolysates contain the mixed sugars that are primarily glucose, xylose, and arabinose. Therefore, these three individual sugars were used, separately, as co-substrates with glycerol, in 1,3-PD production by a *Clostridium diolis* strain DSM 15410, resulting in an 18%–28% increase in the 1,3-PD yield. Co-fermentation of the mixed sugars and glycerol obtained a higher intracellular NADH/NAD^+^ ratio and increased the 1,3-PD yield by 22% relative to fermentation of glycerol alone. Thereafter, two kinds of lignocellulosic hydrolysates, corn stover hydrolysate and corncob molasses, were individually co-fermented with glycerol. The maximum 1,3-PD yield from glycerol reached 0.85 mol/mol. Fed-batch co-fermentation was also performed, improving the 1,3-PD yield (from 0.62 mol/mol to 0.82 mol/mol). These results demonstrate that the co-fermentation strategy is an efficient and economical way to produce 1,3-PD from glycerol.

Driven by energy insecurity and environmental concerns, the sustainable production of chemicals, fuels, and materials has drawn increasing attention recently^1^. To provide these sustainable solutions, exploration of renewable bioresources and development of bio-refineries are required^2^,^3^. The platform chemical 1,3-propanediol (1,3-PD) is commonly used as a monomer for the synthesis of novel polyesters and biodegradable plastics, such as polytrimethylene terephthalate (PTT). Because PTT has superior stretching and stretch recovery characteristics, the production of PTT has caused a drastic rise in the demand of 1,3-PD^1^. The market demand for 1,3-PD was over 60 kt in 2012, and is expected to increase to approximately 150 kt by 2019^4^.

Glycerol is metabolized in a dismutation process via two branches: reductive and oxidative. The reductive branch leads to 1,3-PD production with the consumption of NADH. Meanwhile, glycerol is oxidized to such metabolites as CO_2_, H_2_, acetate, butyrate, lactate, ethanol, butanol, or 2,3-butanediol, producing energy and reducing power for cell growth and 1,3-PD production^5^. Therefore, when glycerol is utilized as the sole carbon source, the conversion yield of 1,3-PD from glycerol is usually below 0.72 mol/mol (the theoretical yield)^6^. In the case of the production of a bulk chemical like 1,3-PD, the cost of the product is mostly affected by the prices of raw materials^7^. For the low yield of 1,3-PD from glycerol, low-cost raw materials and high conversion yield are essential for economical production. Co-fermentation of glycerol and another cheap substrate may help decrease the cost of 1,3-PD production^8^. Since lignocellulose is the most abundant biomass on earth, many efforts have been devoted to develop cost-effective depolymerization techniques^3^,^9^. Lignocellulosic hydrolysates that contain sugars including glucose, xylose and arabinose have become low-cost, accessible and renewable bioresources, which are considered as alternative substrates for bioproduction^10^. Therefore, co-fermentation of glycerol and lignocellulosic hydrolysates could be a valuable approach to reduce the 1,3-PD production costs.

Non-pathogenic *Clostridium* sp., such as *C. butyricum* and *C. diolis*, have been regarded as good producers of 1,3-PD, in terms of ability to tolerate substrates, yield, and productivity^11^. Compared to aerobic or microaerobic fermentation by *Klebsiella* sp., *Clostridium* sp. use the coenzyme B_12_-independent 1,3-PD biosynthetic pathway and can convert glycerol into 1,3-PD anaerobically, making the production process more economically feasible^12^. However, no genetic tools have been available for *C. butyricum* and *C. diolis*^5^,^13^, making the metabolic engineering of these strains difficult. Glycerol fermentation with cheap co-substrates opens the possibility to further improve 1,3-PD production. However, co-fermentation of glycerol and lignocellulosic hydrolysates for 1,3-PD production by *Clostridium* has not been reported yet, and the effects of pentose on glycerol metabolism in *Clostridium* have also not been investigated.

In the present study, for effective and low-cost 1,3-PD production, the potential use of lignocellulosic hydrolysates as co-substrates by *C. diolis* DSM 15410 was investigated. First, we examined the effects of glucose, xylose, and arabinose addition on glycerol metabolism and 1,3-PD biosynthesis. Then, two kinds of lignocellulosic hydrolysates, corn stover hydrolysate and corncob molasses, were selected as the co-substrates, and the ratio of sugars to glycerol was optimized. By these means, the yield of 1,3-PD from glycerol was elevated. The effects of sugar addition on the intracellular NADH/NAD^+^ ratio and mRNA levels of the key genes involved in glycerol metabolism were also investigated.

## Results and Discussion

### Co-fermentation of glycerol and glucose, xylose, or arabinose

**Figure 1:**
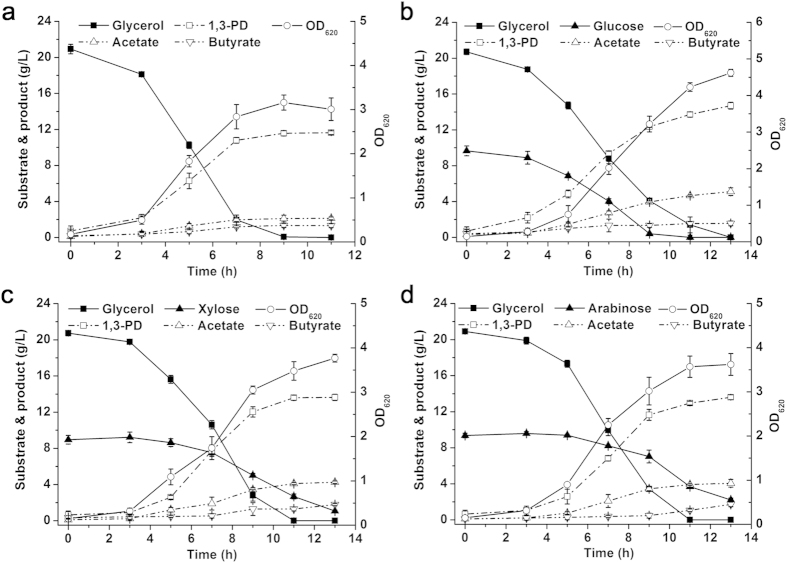
Effects of sugar addition on glycerol metabolism and 1,3-PD production by *C. diolis* DSM 15410. Batch fermentation was carried out at 34 °C in a 5-L bioreactor, and the pH was maintained at 6.8 by automatic addition of 2 M KOH. The culture was stirred at 100 rpm and sparged with nitrogen at a flow rate of 0.1 volume of gas per volume of liquid per minute. (**a**) Glycerol was used as substrate. (**b**) Glucose and glycerol were used as co-substrates. (**c**) Xylose and glycerol were used as co-substrates. (**d**) Arabinose and glycerol were used as co-substrates. The initial concentration of glycerol was approximately 20.0 g/L, and the concentration of refined sugar was approximately 10.0 g/L. The error bars indicate the standard deviation among the three parallel replicates.

Lignocellulosic hydrolysates, such as corn stover hydrolysate and corncob molasses, mainly contain glucose, xylose, and arabinose. Only the effect of glucose addition on glycerol metabolism in *Clostridium* has been reported^14^. Therefore, glycerol-glucose, glycerol-xylose, and glycerol-arabinose co-utilization by *C. diolis* DSM 15410 was first carried out to investigate the effects of sugar addition on glycerol utilization and 1,3-PD production. Batch fermentations were performed using glycerol or glycerol and sugar as carbon source in a 5-L fermentor with an initial glycerol concentration of approximately 20.0 g/L (Fig. 1). When used as the sole carbon source, glycerol (20.0 g/L) was used up in 9 h, and 11.6 g/L of 1,3-PD and a small amount of acetate and butyrate were produced (Fig. 1a). When 10.0 g/L of glucose was added as a co-substrate, it was consumed with glycerol simultaneously (Fig. 1b). However, the glycerol uptake rate decreased, especially in the first 3 h, which might be due to the co-utilization of glycerol and glucose. Significant increases in cell growth and acetate accumulation were observed, and the final titer of 1,3-PD increased to 14.7 g/L. These results suggest that glucose could be used as a co-substrate of glycerol fermentation for 1,3-PD production, which is consistent with the findings of previous studies^14^,^15^,^16^,^17^.

Glycerol-xylose and glycerol-arabinose co-fermentations were then conducted to investigate the effects of a pentose on glycerol metabolism. As shown in Fig. 1c, glycerol and xylose were metabolized simultaneously, and 1,3-PD production was increased, which was similar to glycerol-glucose co-fermentation. However, compared to glycerol-glucose co-fermentation, a relatively faster consumption rate of glycerol was observed. A similar phenomenon was observed when arabinose was added as the co-substrate (Fig. 1d). The results suggest that an addition of pentose has less impact on the glycerol metabolism than glucose, which may be because *C. diolis* DSM 15410 has no obvious preference between pentose and glycerol, and co-fermentation of pentose and glycerol is, however, helpful in the regard of improving the 1,3-PD yield.

The conversion yields of 1,3-PD from glycerol in glycerol fermentation and glycerol-sugar co-fermentation were calculated and the results are shown in Table 1. When glucose, xylose, and arabinose were co-utilized with glycerol, the yield of 1,3-PD to glycerol increased by 28% (0.86 mol/mol), 19% (0.80 mol/mol), and 18% (0.79 mol/mol), respectively. This demonstrated that adding sugar as a co-substrate, whether it was a hexose or pentose, would be a feasible way to improve the 1,3-PD yield. Furthermore, the biomass of co-fermentation also increased, which would be advantageous to 1,3-PD production.

An analysis of by-product formation suggested that acetate and butyrate production both increased when sugar was co-utilized with glycerol (Table 1). In particular, acetate production more than doubled. It has been reported that when *C. butyricum* used glucose as the sole substrate, butyrate was the main product. However, glucose metabolism shifted from butyrate to acetate production in the presence of glycerol, and more reducing power and ATP were available for cell growth and reduction of glycerol^14^,^15^. It was suggested that the addition of a pentose, such as xylose and arabinose, would have the same effects. The increasing acetate formation led to more NADH and ATP production. Overall, all these results confirmed the feasibility that simultaneous glycerol-sugar consumption could increase the production and conversion yield of 1,3-PD.

Using the data in Table 1, NADH generation and consumption were calculated to check the NADH balance of the fermentation (Table S1). When glycerol or glycerol-sugar mixture was utilized by *Clostridium*, a large part of the reduced ferredoxin that was formed during oxidation of pyruvate into acetyl-CoA was reoxidized by the ferredoxin-NAD(P) reductase to produce NADH, and the rest was reoxidized by the hydrogenase to produce H_2_^6^,^18^. In this study, considering the maximum and minimum hydrogen production, the values of NADH generation were calculated (Table S1). The value of NADH consumption was between the maximum and minimum values of NADH generation, which was reasonable for the anaerobic 1,3-PD production by *Clostridium*.

### Co-fermentation of glycerol and ^13^C-labeled glucose

**Figure 2:**
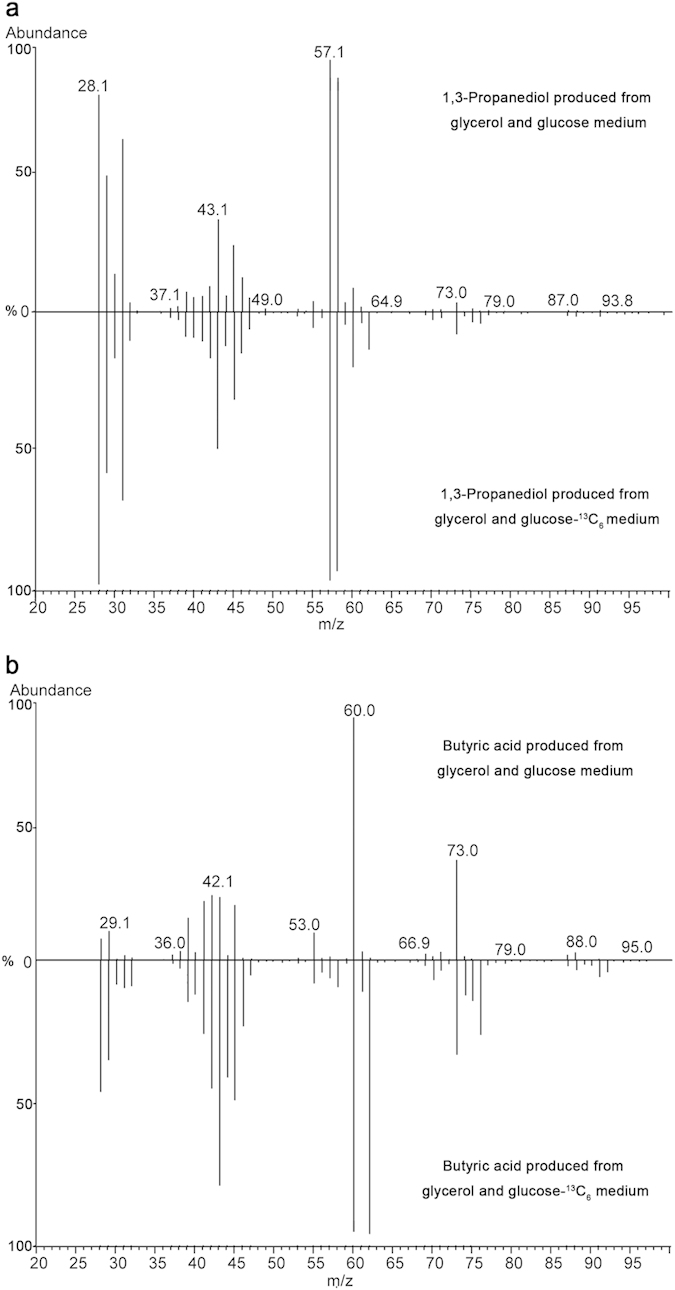
Detection of 1,3-PD and butyric acid produced by *C. diolis* DSM 15410 from glycerol and glucose (or glucose-^13^C_6_) medium using GC-MS. (**a**) 1,3-PD. (**b**) Butyric acid.

To verify if the carbon skeleton of 1,3-PD was originated from sugars, co-fermentation of glycerol and glucose-^13^C_6_ (all six carbon atoms in the glucose were ^13^C labeled) was also carried out. The mass spectrum of 1,3-PD produced from the glycerol and glucose-^13^C_6_ medium matches well with that from glycerol and glucose medium (Fig. 2a). Thus, there was no ^13^C-labeled 1,3-PD; however, there was ^13^C-labeled butyric acid detected when glycerol and glucose-^13^C_6_ medium was used (Fig. 2b). The results suggest that 1,3-PD was not produced from glucose but only from glycerol. Thus far, no microorganism capable of converting sugars such as glucose and xylose directly to 1,3-PD has been discovered due to the absence of glycerol-3-phophatase that catalyzes the reaction from glycerol-3-phosphate to glycerol^19^.

### Co-fermentation of glycerol and mixed sugars

**Figure 3:**
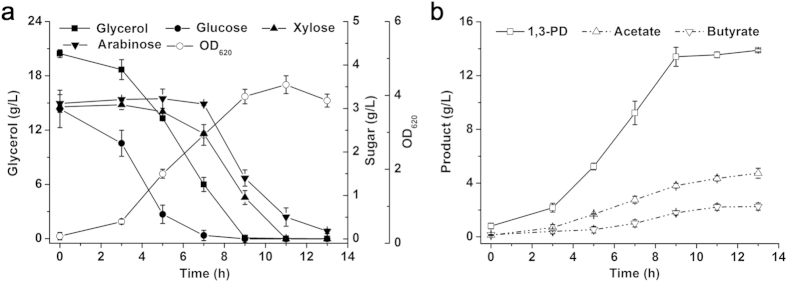
Effects of addition of the mixed sugars on glycerol metabolism and 1,3-PD production by *C. diolis* DSM 15410. Batch fermentation was carried out at 34 °C in a 5-L bioreactor, and the pH was maintained at 6.8 by automatic addition of 2 M KOH. The culture was stirred at 100 rpm and sparged with nitrogen at a flow rate of 0.1 volume of gas per volume of liquid per minute. The initial concentration of glycerol is approximately 20.0 g/L. Mixed sugars (glucose:xylose:arabinose = 1:1:1) were added as co-substrates. The error bars indicate the standard deviation among the three parallel replicates.

Hydrolysis of lignocellulose generates a mixture of sugars containing primarily glucose, xylose, and arabinose. Although all three of these sugars can be concurrently utilized with glycerol at a specific rate during fermentation and distinctly enhance the 1,3-PD yield from glycerol, the influence of combined sugars on glycerol metabolism by *C. diolis* DSM 15410 has not been characterized. Therefore, batch fermentation was performed using 20.0 g/L of glycerol and 10.0 g/L of mixed sugars including glucose, xylose, and arabinose (mass ratio = 1:1:1) as carbon sources. As shown in Fig. 3a, glucose was utilized preferentially, while xylose and arabinose were used at the moment when glucose was almost completely depleted. The rapid glucose utilization rate is not surprising because of the carbon catabolite repression effect. The arabinose consumption rate was a little slower than that of xylose. All sugars were exhausted after 11 h. It is worth noting that glycerol was metabolized throughout the fermentation process, and that glycerol utilization was only minimally inhibited by the mixed sugars.

Co-fermentation of glycerol and the mixed sugars also resulted in a higher production and conversion yield of 1,3-PD. After 13 h, the final titer and conversion yield of 1,3-PD reached 13.9 g/L and 0.82 mol/mol (Fig. 3b), which were 19% and 22% higher, respectively, than those from glycerol fermentation alone. The 1,3-PD production profiles confirmed the simultaneous uptake and metabolism of glycerol and sugars included in lignocellulosic hydrolysates. Since the proportion of lignocellulose-derived sugars is diverse, this feature of *C. diolis* DSM 15410 is essential for the economical feasibility of the biorefinery process. Moreover, acetate was still the main by-product, which would lead to increased NADH availability and further favor 1,3-PD biosynthesis. Although the mixed sugars could not be converted to 1,3-PD, the enhanced cell growth and reducing power markedly improved 1,3-PD production^8^.

### Effects of mixed sugars on *dhaB1*, *dhaD*, and *dhaT* mRNAs levels

**Figure 4:**
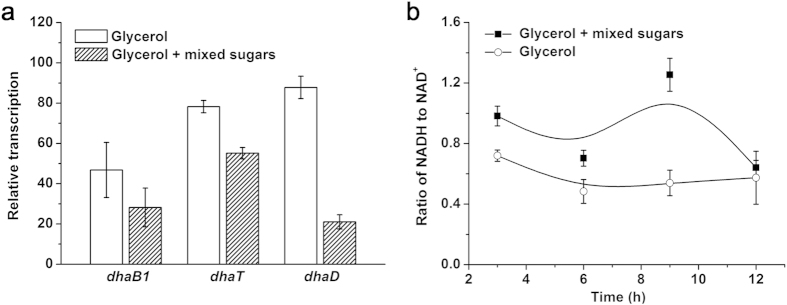
Effects of the mixed sugars on transcriptional levels of key genes involved in glycerol metabolism and internal redox reducing equivalent in *C. diolis* DSM 15410. (**a**) Determination of relative transcriptional levels of *dhaB1*, *dhaT*, and *dhaD* using quantitative PCR. (**b**) Determination of intracellular NADH/NAD^+^ ratio. Cells were cultured using glycerol and mixed sugars (glucose:xylose:arabinose = 1:1:1) as co-carbon sources. Samples were collected in the middle of the exponential phase when OD_620_ was approximately 1.0. At the same time, cells cultured in glycerol medium were taken as control. The initial glycerol concentration was 20.0 g/L and the total sugars concentration was 10.0 g/L. The error bars indicate the standard deviation among the three parallel replicates.

During the co-fermentation process, no apparent inhibition of glycerol utilization and 1,3-PD production was observed, suggesting that the addition of glucose, xylose, and arabinose mixtures did not repress the glycerol metabolism pathway significantly. Glycerol dehydratase (GDHt), 1,3-PD dehydrogenase (1,3-PDDH), and glycerol dehydrogenase (GDH), which are encoded by *dhaB1*, *dhaT*, and *dhaD*, respectively, are key enzymes involved in glycerol metabolism in *Clostridium*^20^. To the best of our knowledge, no study has evaluated the transcriptional regulation of these genes from *Clostridium* when glycerol was co-fermented with sugar mixtures. Therefore, quantitative reverse transcription-PCR was performed to determine the transcription levels of *dhaB1*, *dhaT*, and *dhaD*. As shown in Fig. 4a, the transcription levels of *dhaB1* and *dhaT* were slightly affected, whereas the mRNA level of *dhaD* decreased by ~3 fold with the addition of mixed sugars, suggesting that glycerol oxidation was inhibited more significantly than glycerol reduction. Malaoui and Marczak studied the influence of glucose as a co-substrate in glycerol fermentation on 1,3-PD in *C. butyricum* E5^16^. The assay of specific enzymatic activities demonstrated that the GDH activity was progressively decreased with increasing glucose inputs^16^, which was similar to the results of our study.

### Effect of mixed sugars on the intracellular level of NADH/NAD^+^

Cofactor manipulations have been reported as an efficient strategy for improving the production of reduced chemicals like 1,3-PD and 2,3-butanediol^21^,^22^. Biosynthesis of 1,3-PD from glycerol in *Clostridium* is NADH-dependent, making the supply of NADH important for efficient 1,3-PD production. In the present study, the addition of an economically attractive carbon source as a co-substrate can feasibly increase 1,3-PD yield from glycerol. The by-product analysis also demonstrated that more acetate was produced when sugars were co-utilized, which was beneficial for NADH regeneration. Therefore, we speculated that the intracellular NADH pool had changed, and determined the intracellular concentrations of NADH and NAD^+^. As shown in Fig. 4b, compared to glycerol as the only carbon source, the addition of a sugar mixture as co-substrates resulted in a higher ratio of intracellular NADH/NAD^+^. Moreover, variations in the 1,3-PD production rate basically followed the NADH/NAD^+^ ratio, which suggested that NADH/NAD^+^ levels played a crucial role in NADH-dependent pathways such as 1,3-PD synthesis. This result demonstrates that co-fermentation of glycerol and sugar mixtures contained in lignocellulosic hydrolysates can efficiently increase the level of intracellular reducing equivalents, which can further increase the 1,3-PD conversion yield.

### Optimization of the ratio of glycerol to lignocellulosic hydrolysate

Since *C. diolis* DSM 15410 can co-utilize glycerol with hexoses and pentoses, using lignocellulosic hydrolysates as co-substrates for 1,3-PD production is viable. Thus, two kinds of lignocellulosic hydrolysates, corn stover hydolysate and corncob molasses, were selected as co-substrates for glycerol fermentation. To investigate the effect of total sugar concentration on 1,3-PD production and save the cost of substrates, the ratios of glycerol to total sugars of corn stover hydolysate and corncob molasses were optimized. When corn stover hydolysate was added at a glycerol to total sugar ratio of 8:1, the cell growth and 1,3-PD yield both increased (Table 2), indicating that small amounts of corn stover hydolysate can facilitate 1,3-PD production. As the concentration of corn stover hydolysate increased, the cell growth increased accordingly. However, the 1,3-PD yield reached its maximum (0.84–0.85 mol/mol) when the ratio of glycerol to total sugars was 4:1. Further increasing the total sugar concentration only promoted the production of butyrate noticeably, which was unfavorable to 1,3-PD synthesis due to NADH generation. Similar results were achieved with corncob molasses as a co-substrate, and the maximum 1,3-PD yield was obtained when the ratio of glycerol to total sugars was 4:1 (Table 2). Based on these results, a ratio of 4:1 (glycerol to total sugars) was selected for the subsequent experiments.

### Fed-batch fermentation of corn stover hydrolysate as co-substrate

In order to avoid substrate inhibition and improve the final titer of target products, fed-batch fermentation is often carried out. To check whether the co-utilization strategy of glycerol-sugars was effective in fed-batch fermentation, *C. diolis* DSM 15410 was cultured using glycerol (20.0 g/L) and corn stover hydrolysate (5.0 g/L of total sugars) as initial carbon sources. A mixture containing glycerol and corn stover hydrolysate (glycerol:total sugars = 4:1) was added into the culture the moment glycerol was almost exhausted. In addition, in order to test whether the accumulation of inhibitors in the hydrolysates is inhibitory to 1,3-PD production, fed-batch fermentation using glycerol and mixed sugars mimicking the sugar profile of corn stover hydrolysate as co-substrates was performed. Control experiment using pure glycerol as the only carbon source was also carried out.

**Figure 5:**
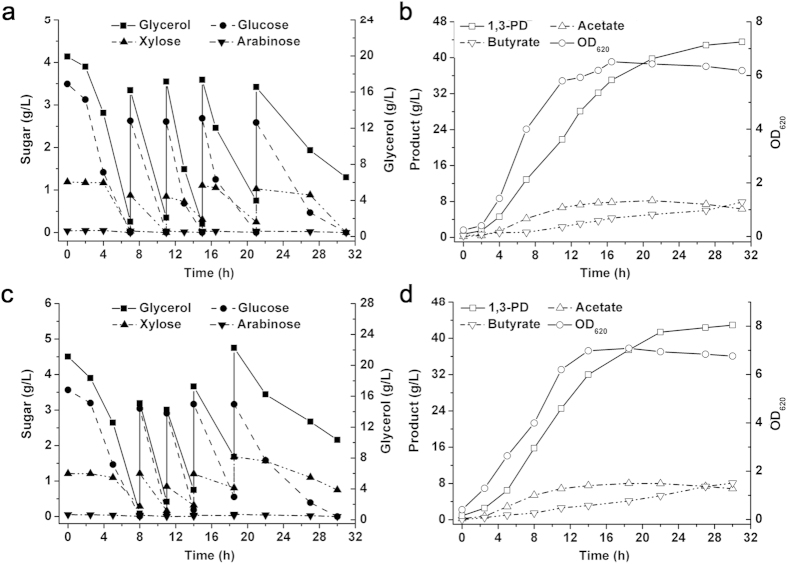
1,3-PD production using co-substrates by *C. diolis* DSM 15410 in fed-batch fermentation. Fermentation was carried out at 34 °C in a 5-L bioreactor, and the pH was maintained at 6.8 by automatic addition of 2 M KOH. The culture was stirred at 100 rpm and sparged with nitrogen at a flow rate of 0.1 volume of gas per volume of liquid per minute. (**a,b**) Glycerol and corn stover hydrolysate were used as co-substrates. (**c,d**) Crude glycerol and corn stover hydrolysate were used as co-substrates. The initial concentration of glycerol was approximately 20.0 g/L, and the concentration of total sugars was approximately 5.0 g/L. A mixture containing glycerol (or crude glycerol) and corn stover hydrolysate (glycerol:total sugars = 4:1) was added separately in the culture the moment glycerol was almost exhausted.

Considering glycerol utilization, cell growth and metabolite profiles, fed-batch fermentation using glycerol and corn stover hydrolysate can be divided into three periods (Fig. 5a,b). During Period I (0–2 h), cells were in the lag phase, when the cell growth and substrate utilization were both slow. Period II (2–16.5 h), the exponential phase, is the stage when glycerol and corn stover hydrolysate were metabolized rapidly and the cell biomass increased steadily. During this period, 1,3-PD was mainly produced, with a productivity of 2.3 g/L·h. The synthesis of 1,3-PD was accomplished with the accumulation of acetate and butyrate, among which acetate was the main by-product. Then, the fermentation process entered into Period III (16.5–31 h), the stationary phase, when the cell growth ceased, and the rates of substrate utilization and 1,3-PD production decreased dramatically. The production of acetate was also hindered and acetate was re-utilized as its concentration decreased. The re-utilization of acetate was also observed in previous studies on fermentation by *Clostridium*^23^,^24^,^25^. On the contrary, butyrate was synthesized steadily. After 31 h, 43.5 g/L of 1,3-PD was obtained with a high conversion yield of 0.82 mol/mol.

When glycerol and mixed sugars mimicking the sugar profile of corn stover hydrolysate were co-fermented, similar 1,3-PD concentration (45.4 g/L) and conversion yield (0.82 mol/mol) were achieved (Fig. S1). The results suggest that the inhibitors in corn stover hydrolysate don’t have obvious negative effects on 1,3-PD biosynthesis. Moreover, fed-batch fermentation of pure glycerol can also obtain a similar titer of 1,3-PD (43.9 g/L) (Fig. S2), but the conversion yield from glycerol to 1,3-PD was only 0.62 mol/mol. Therefore, from the perspective of conversion yield, the 1,3-PD production was obviously enhanced by the co-fermentation strategy.

As shown in Fig. 5a,b, the productivity decreased during Period III, and the final titer of 1,3-PD was not very satisfactory, probably for two reasons. First, the production of 1,3-PD required NADH as a cofactor, which was generated through the oxidation of glycerol or sugars. In Period II of the fed-batch fermentation process, glycerol and sugars were rapidly oxidized with the production of NADH. Co-utilization of glycerol and corn stover hydrolysate provided a strong driving force for the conversion of glycerol into 1,3-PD. However, cells in the stationary phase metabolized substrates more slowly. Without a sufficient supply of NADH, the efficiency of 1,3-PD was greatly reduced.

Second, glycerol fermentation usually results in a low concentration of 1,3-PD due to inhibition of products such as acetate and 1,3-PD^1^,^26^,^27^,^28^,^29^. Utilization of corn stover hydrolysate led to more acetate production, which should be beneficial for 1,3-PD production since synthesis of acetate provided the maximum supply of NADH. However, the accumulation of acetate ceased when its concentration reached approximately 8.0 g/L; accordingly, 1,3-PD production slowed. Colin *et al.* reported that the initial addition of acetate favored cell growth and butyrate production, but reduced 1,3-PD production in *C. butyricum*^27^. Acetate was even determined to be the most toxic product among the acids excreted during the glycerol fermentation process^30^. Additionally, it has been suggested that self-produced acetate was more toxic to *C. butyricum* than acetate that was externally added^26^. Thus, the high concentration of acetate obtained in the present study might be responsible for the inhibition of 1,3-PD production, especially in Period III. Meanwhile, the inhibitory effect of 1,3-PD has also been investigated in previous studies. The accumulation of 1,3-PD could inhibit the cell growth of *C. butyricum* distinctly. Screening the product tolerant mutants could help to obtain a higher titer of 1,3-PD^28^,^29^.

To further decrease the cost of 1,3-PD production, fed-batch fermentation of crude glycerol and corn stover hydrolysate was carried out (Fig. 5c,d). At the end, 42.9 g/L 1,3-PD was obtained, and the final conversion yield was 0.81 mol/mol, which was 31% higher than that from glycerol alone. In any case, the high PD yield achieved by co-fermentation strategy is satisfied compared with previous studies (Table 3). These results demonstrate that utilization of lignocellulosic hydrolysates as co-substrates is an efficient and economical way to biosynthesize 1,3-PD. Continuous co-fermentation of glycerol and lignocellulosic hydrolysates may help to bypass product inhibition and achieve long-term and highly efficient 1,3-PD production. The co-utilization strategy presented here could also be applied to develop economical production processes of other valuable chemicals.

## Methods

### Lignocellulosic hydrolysates

The corn stover hydrolysate was kindly provided by Changchun Dacheng Group Co., Ltd. (Changchun, China) with a sugar content of 411.0 g/L glucose, 140.8 g/L xylose and 5.0 g/L arabinose^10^. The hydrolysis technique includes steam explosion, stewing, enzymatic hydrolysis, and concentration. Corncob molasses, a waste by-product in xylitol production, was supplied by Longlive Bio-technology Co., Ltd. (Shandong, China) and contained 110.0 g/L glucose, 352.9 g/L xylose and 146.3 g/L arabinose^31^,^32^. It was produced from corncob by steam explosion, stewing, enzymatic hydrolysis, concentration, crystallization of xylose, and filtration. Xylose is then used to produce xylitol while the residual hydrolysate, corncob molasses, could be used as a cheap feedstock for bio-production.

### Bacterial strain and growth conditions

*C. diolis* DSM 15410 was obtained from the German Collection of Microorganisms (DSM). Spores were maintained at 4 °C in Reinforced Clostridial Medium (RCM; Oxoid Ltd, Basingstoke, UK) in screw-capped bottles. The synthetic medium used contained the following components (L^−1^ distilled water): glycerol, 20 g; K_2_HPO_4_·3H_2_O, 1.3 g; KH_2_PO_4_, 0.5 g; (NH_4_)_2_SO_4_, 2.0 g; MgSO_4_, 0.1 g; CaCl_2_, 0.1 g; yeast extract, 1.0 g; Fe solution (FeSO_4_·7H_2_O, 5.0 g/L; 37% HCl), 1 mL; trace element solution, 2 mL^33^. To limit pH variations during fermentation, 2.0 g/L CaCO_3_ was added. The cultures were grown in 50 mL screw-capped bottles with butyl rubber stoppers for syringe operation. After being filled with 40 mL synthetic medium, the bottles were sterilized at 121 °C for 20 min, and 0.02% sodium hyposulfite was added before inoculation. Four milliliters of cell suspension was inoculated into 40 mL medium and incubated anaerobically for 14–24 h at 34 °C before fermentation studies. Growth of *C. diolis* DSM 15410 was monitored by measuring the optical density at 620 nm (OD_620_)^13^,^34^.

### Co-fermentation of glycerol and sugars

The co-fermentation medium was based on the synthetic medium, and was supplemented with sugar, mixture of sugars, or lignocellulosic hydrolysate to the desired initial concentration. Fermentation was conducted in a 5-L stirred bioreactor (BIOSTAT B, B. Braun Biotech International GmbH, Germany) with 2 L of initial medium that was inoculated with 10% (v/v) of seed cultures. All fermentations were carried out at 34 °C, and the pH was maintained at 6.8 by automatic addition of 2 M KOH. The culture was stirred at 100 rpm and sparged with nitrogen at a flow rate of 0.1 volume of gas per volume of liquid per minute. Antifoam (Polyoxyethylenepolyoxypropylenepolyol ethers, 100 μL/2 L medium) was added to the medium before sterilization in case of foaming during the fermentation course. To examine the effect of sugars on glycerol metabolism, 10.0 g/L of sterilized glucose, xylose, arabinose, or mixture of the three sugars (mass ratio = 1:1:1) was added in the cultures before inoculation. To verify if the carbon skeleton of 1,3-PD was originated from glucose, ^13^C labeling experiment using 20.0 g/L glycerol and 10.0 g/L glucose-^13^C_6_ (all six carbon atoms in the glucose were ^13^C labeled from Sigma) as the co-substrates was performed. To study the optimal initial concentration ratio of total sugars in lignocellulosic hydrolysates to glycerol, batch fermentations were performed using the basic medium with the initial total sugar concentrations of approximately 2.5, 5.0, 10.0, and 20.0 g/L. Among all the batch fermentations above, 20.0 g/L of glycerol was added initially. In fed-batch fermentation, the initial glycerol concentration was approximately 20.0 g/L, and mixture of glycerol and corn stover hydrolysate (or mixed sugars mimicking the sugar profile of corn stover hydrolysate) (ratio of glycerol to total sugars approximately 4:1) was added when glycerol was almost used out in the culture. Samples were withdrawn periodically. The biomass, concentrations of residual substrates, 1,3-PD, and by-products were detected afterwards.

### Fed-batch fermentation of crude glycerol and corn stover hydrolysate

The composition of the crude glycerol was as follows (percent weight per weight): glycerol, 80%; moisture content, 9%; ash content, 5%; MONG (matter organic non-glycerol), 0.9%; methanol, 0.1%. Crude glycerol concentrations given in this study will state the absolute glycerol content of the solution irrespective of the impurities. The operational procedure was same with the methods described above.

### Quantitation of *dhaB1*, *dhaD* and *dhaT* mRNAs

Cells of *C*. *diolis* DSM 15410 cultured using glycerol and mixed sugars (mass ratio = 1:1:1) as co-carbon sources were quickly collected in the middle of the exponential phase when OD_620_ was approximately 1.0. Cells incubated in pure glycerol medium were collected at the same time and taken as control. Total RNA was isolated using the RNAprep pure Cell/Bacteria Kit (Tiangen Biotech, Beijing, China) and then treated with DNase I (MBI, Fermentas, Lithuania). After that, cDNA was synthesized subsequently using random primes, SuperScript III Reverse Transcriptase (Invitrogen), and RNA as a template. Primes for *dhaB1*, *dhaD*, *dhaT*, and 16S rRNA were designed with Beacon Designer software. The primer sequences for *dhaB1*, *dhaD*, *dhaT*, and 16S rRNA were 5′-ACCTCAACCATCTCTATCAGTAAG-3′ (forward), and 5′-GGCTCAACACATCCAATTATTCC-3′ (reverse); 5′-CTTGGTGGTGGTAAAGCGATTG-3′ (forward), and 5′-TGGTGTGTAAAGAACTGCTGAATG-3′ (reverse); 5′-CTGTAGGTGGAGGGAGTTC-3′ (forward), 5′-AGTTAATACACAATGACGAGTTAC-3′ (reverse); 5′-GCGGAATACTTAATGCGTTAGC-3′ (forward), and 5′-TGCGTAGAGATTAGGAAGAATACC-3′ (reverse), respectively. Quantitative PCR analysis was then carried out by using SuperReal Premix SYBR Green kit (Tiangen Biotech, Beijing, China) and the CFX96 Real-time system (Bio-Rad) according to the manufacturers’ instructions.

### Determination of the reducing equivalent

The intracellular concentrations of NADH and NAD^+^ were determined using the NAD^+^/NADH Quantification Colorimetric kit from BioVision (Mountain View, CA, USA) according to the manufacturer’s instructions. About 2 × 10^6^ cells were collected for each assay and extracted immediately with 400 μL of NADH/NAD^+^ Extraction Buffer by two freeze/thaw cycles. The assay kit is based on the NAD^+^ Cycling Enzyme Mix which can specifically recognize NADH/NAD^+^ in an enzyme cycling reaction. The reaction is performed in labeled 96-well plate and measured at OD_450_ after adding Stop Buffer. The value of NADH/NAD^+^ can be quantified by comparing with standard NADH.

### Analytical techniques

1,3-PD and butyrate produced by *C. diolis* DSM 15410 from glycerol and glucose-^13^C_6_ medium were detected by gas chromatography-mass spectrometry (GC-MS) analysis. Prior to GC-MS, samples were extracted with an equal volume of ethyl acetate. Gas mass spectra (EI-MS) was recorded on GC-MS spectrometers with HP-5 ms columns (0.25 μm, 0.25 μm × 30 m) (Agilent Technologies 6850/5975C, USA, for EI-MS). Helium was used as the carrier gas. The injector and detector temperatures were maintained at 260 °C. The GC oven temperature was maintained at 120 °C. The column flow rate was maintained at 0.5 ml/min. Concentrations of 1,3-PD, glycerol, sugars, and organic acids such as acetate and butyrate, were quantified by high-performance liquid chromatography (HPLC) (Agilent 1100 series, Hewlett-Packard) with the method described by Wang *et al.*^31^. The HPLC system was equipped with a refractive index detector and a Bio-Rad Aminex HPX-87H column (300 × 7.8 mm). The analysis was performed with a mobile phase of 5 mM H_2_SO_4_ at 55 °C with a flow rate of 0.5 mL/min. The injection volume was 10 μL. Samples were centrifuged at 12,000 × *g* for 10 min. The supernatant was used for detection. The concentrations of chemicals above were estimated with calibration curves. The yield of 1,3-PD from glycerol was defined as 1,3-PD produced (mol)/glycerol consumed (mol).

## Additional Information

**How to cite this article**: Xin, B. *et al.* Co-utilization of glycerol and lignocellulosic hydrolysates enhances anaerobic 1,3-propanediol production by *Clostridium diolis*. *Sci. Rep.* **6**, 19044; doi: 10.1038/srep19044 (2016).

## Supplementary Material

Supplementary Information

## Figures and Tables

**Table 1:** Effect of sugar addition on glycerol metabolism in *C. diolis* DSM 15410.

Carbon source	Carbon consumed (g/L)		Product (g/L)			Biomass (OD_620_)	Yield (mol 1,3-PD/mol Glycerol)
	Glycerol	Sugar	1,3-PD	Acetate	Butyrate		
Glycerol	20.93 ± 0.23	0	11.64 ± 0.21	2.13 ± 0.16	1.32 ± 0.22	3.01 ± 0.25	0.67
Glycerol + glucose	20.71 ± 0.32	9.65 ± 0.55	14.70 ± 0.38	5.11 ± 0.43	1.56 ± 0.27	4.62 ± 0.09	0.86
Glycerol + xylose	20.73 ± 0.30	8.15 ± 0.59	13.64 ± 0.35	4.25 ± 0.14	1.78 ± 0.12	3.77 ± 0.09	0.80
Glycerol + arabinose	20.91 ± 0.11	7.16 ± 0.23	13.60 ± 0.27	4.06 ± 0.43	1.71 ± 0.26	3.62 ± 0.25	0.79

**Table 2:** Optimization of the ratio of glycerol to sugars in lignocellulosic hydrolysates for 1,3-PD production.

Co-substrate	Glycerol (g): sugars (g)	Carbon consumed (g/L)		Product (g/L)			Biomass (OD_620_)	Yield (mol 1,3-PD/mol Glycerol)
		Glycerol	Sugar	1,3-PD	Acetate	Butyrate		
Corn stover hydolysate	1:1	19.91 ± 0.27	20.05 ± 0.25	14.03 ± 0.17	4.26 ± 0.38	5.70 ± 0.12	5.85 ± 0.05	0.85
	2:1	20.79 ± 0.18	10.11 ± 0.14	14.58 ± 0.07	4.68 ± 0.25	3.26 ± 0.06	5.30 ± 0.16	0.85
	4:1	20.35 ± 0.22	4.88 ± 0.30	14.21 ± 0.15	3.93 ± 0.37	1.40 ± 0.21	4.04 ± 0.28	0.84
	8:1	19.30 ± 0.36	2.62 ± 0.09	12.44 ± 0.23	3.29 ± 0.29	1.10 ± 0.11	3.70 ± 0.24	0.78
Corncob molasses	1:1	20.24 ± 0.19	19.64 ± 0.36	13.89 ± 0.08	4.46 ± 0.22	7.11 ± 0.33	4.97 ± 0.35	0.83
	2:1	20.67 ± 0.06	10.39 ± 0.12	14.24 ± 0.15	4.54 ± 0.27	2.85 ± 0.07	4.47 ± 0.26	0.83
	4:1	20.37 ± 0.19	5.18 ± 0.07	14.11 ± 0.22	4.41 ± 0.12	1.42 ± 0.30	4.04 ± 0.08	0.84
	8:1	20.54 ± 0.11	2.49 ± 0.20	12.79 ± 0.16	3.48 ± 0.23	1.38 ± 0.10	3.05 ± 0.28	0.75

**Table 3:** 1,3-PD production using different substrates by *Clostridium*.

Substrate	Strain	Fermentation type	Concentration (g/L)	Productivity (g/L·h)	Yield (mol 1,3-PD/mol Glycerol)	Reference
Pure glycerol	*C. butyricum* VPI 3266	Batch	35.0	0.7	0.65	11
	*C. butyricum* VPI 3266	Fed-batch	65.0	1.2	0.68	11
	*C. butyricum* DSM 5431	Fed-batch	47.5	2.4	0.62	28
	*C. butyricum* DSM 5431 mutant 2/2	Fed-batch	70.4	1.4	0.68	28
	*C. acetobutylicum* DG1 (pSPD5)	Continuous	31.3	1.6	0.64	13
Crude glycerol	*C. butyricum* VPI 1718	Fed-batch	67.9	0.8	0.67	35
	*C. butyricum* F2b	Continuous two-stage	43.5	1.3	0.59	36
	*C. acetobutylicum* DG1 (pSPD5)	Continuous	29.8	1.5	0.61	13
Pure glycerol and glucose	*C. butyricum* E5	Batch	6.6	NA	0.81	16
	*C. butyricum* DSM 5431	Batch	15.5	0.5	0.93	14
	*C. butyricum* VPI 3266	Continuous	16.4	0.8	0.69	18
	*C. diolis* DSM 15410	Batch	14.7	1.1	0.86	This study
Pure glycerol and xylose	*C. diolis* DSM 15410	Batch	13.6	1.0	0.80	This study
Pure glycerol and arabinose	*C. diolis* DSM 15410	Batch	13.6	1.0	0.79	This study
Pure glycerol and corn stover hydrolysate	*C. diolis* DSM 15410	Fed-batch	43.5	1.4	0.82	This study
Crude glycerol and corn stover hydrolysate	*C. diolis* DSM 15410	Fed-batch	42.9	1.4	0.81	This study
