# Supplementary material for: Co-utilization of glycerol and lignocellulosic hydrolysates enhances anaerobic 1,3-propanediol production by Clostridium diolis
Source: Sci Rep. 2016 Jan 11;6:19044. doi: 10.1038/srep19044 (PMC4707465; doi:10.1038/srep19044)
Supplement: Supplementary Information [file srep19044-s1.pdf]

Supplementary Information

Co-utilization of glycerol and lignocellulosic hydrolysates enhances anaerobic 1,3-propanediol production by *Clostridium diolis*

Bo Xin [1,2], Yu Wang [2,3], Fei Tao [2,3], Lixiang Li [2,3], Cuiqing Ma [1,*], Ping Xu [2,3,4,*]

[1]State Key Laboratory of Microbial Technology, Shandong University, Jinan 250100, PR China

[2]State Key Laboratory of Microbial Metabolism, and School of Life Sciences & Biotechnology, Shanghai Jiao Tong University, Shanghai 200240, PR China

[3]Joint International Research Laboratory of Metabolic & Developmental Sciences, Shanghai Jiao Tong University, Shanghai 200240, PR China

[4]Shanghai Collaborative Innovation Center for Biomanufacturing, East China University of Science and Technology, Shanghai 200237, PR China

*Correspondence: pingxu@sjtu.edu.cn (Ping Xu), or macq@sdu.edu.cn (Cuiqing Ma)

Authors emails:

BX: xinbosdu@163.com

YW: wang_yu@sjtu.edu.cn

FT: taofei@sjtu.edu.cn

LL: lilixiang@sdu.edu.cn

CM: macq@sdu.edu.cn

PX: pingxu@sjtu.edu.cn

Table S1. NADH generation and consumption of *Clostridium diolis* DSM 15410 using glycerol or glycerol-sugar as substrates.

Carbon source	Carbon consumed (mM)		Product (mM)			NADH consumption (mM)[a]	NADH generation (mM)	
	Glycerol	Sugar	1,3-PD	Acetate	Butyrate		Min[b]	Max[c]
Glycerol	227.50	0.00	153.16	35.50	15.00	183.16	148.68	223.03
Glycerol + glucose	225.11	53.61	193.42	85.17	17.73	228.88	170.60	309.51
Glycerol + xylose	225.33	54.33	179.47	70.83	20.23	219.93	182.26	318.67
Glycerol + arabinose	227.28	47.73	178.95	67.67	19.43	217.81	176.23	304.12

[a]NADH consumption = [1,3-PD] + [butyrate] × 2

[b]Minimum NADH generation = [glycerol consumption - 1,3-PD] × 2 + [glucose consumption] × 2 (or [pentose consumption] × 1.67)

[c]Maximum NADH generation = [glycerol consumption - 1,3-PD] × 3 + [glucose consumption] × 4 (or [pentose consumption] × 3.33)

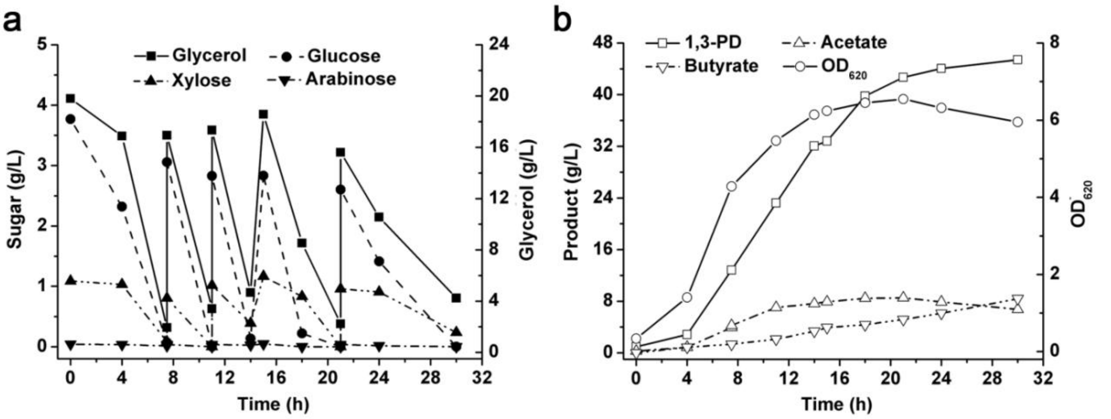

Figure S1. 1,3-PD production using glycerol and mixed sugars mimicking the sugar profile of corn stover hydrolysate as co-substrates by *C. diolis* DSM 15410 in fed-batch fermentation. Fermentation was carried out at 34℃ in a 5-L bioreactor, and the pH was maintained at 6.8 by automatic addition of 2 M KOH. The culture was stirred at 100 rpm and sparged with nitrogen at a flow rate of 0.1 volume of gas per volume of liquid per minute. The initial concentration of glycerol was approximately 20.0 g/L, and the concentration of total sugars was approximately 5.0 g/L. A mixture containing glycerol and mixed sugars (glycerol:total sugars = 4:1) was added in the culture the moment glycerol was almost exhausted.

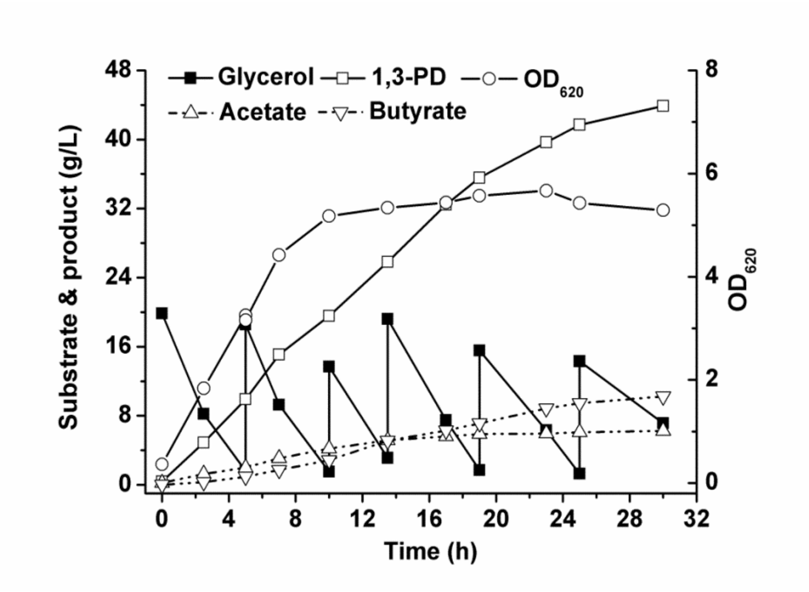

Figure S2. 1,3-PD production using glycerol as the sole substrate by *C. diolis* DSM 15410 in fed-batch fermentation. Fermentation was carried out at 34℃ in a 5-L bioreactor, and the pH was maintained at 6.8 by automatic addition of 2 M KOH. The culture was stirred at 100 rpm and sparged with nitrogen at a flow rate of 0.1 volume of gas per volume of liquid per minute. The initial concentration of glycerol was approximately 20.0 g/L. A total of 40.0 g of glycerol was added in the culture the moment glycerol was almost exhausted.
